# Supplementary material for: IL-1 Superfamily Member (IL-1A, IL-1B and IL-18) Genetic Variants Influence Susceptibility and Clinical Course of Mediterranean Spotter Fever
Source: Biomolecules. 2022 Dec 17;12(12):1892. doi: 10.3390/biom12121892 (PMC9816934; doi:10.3390/biom12121892)
Supplement: Supplementary file 1 [file biomolecules-12-01892-s001.zip › biomolecules-2000949-supplementary.pdf]

Table S1. Analysis of SNP frequencies in patients affected by Mediterranean Spotted Fever (MSF) complicated with Acute Respiratory Distress Syndrome (ARDS) compared to MSF without ARDS (w/oARDS) and Healthy Controls (adjusted by 64 years Age cut off and Gender).

**Genes and SNP Alleles**		**ARDS**		**w/oARDS**		**Controls**		**ARDS Vs w/oARDS**		**ARDS Vs Controls**	
		**N.**	**Freq.**	**N.**	**Freq.**	**N.**	**Freq.**	**OR (95% CI)**	***p* Value**	**OR (95% CI)**	***p* Value**
TIRAP rs8177374	C/C	12	0.80	122	0.84	178	0.86	0.75 (0.20-2.88)	0.713	0.63 (0.17-2.37)	0.448
	C/T	3	0.20	20	0.14	22	0.11	1.66 (0.42-6.57)	0.49	2.24 (0.56-8.98)	0.28
	T/T	0	-	3	0.02	6	0.03	--	--	--	--
*IL-18 * rs187238*	C/C	2	0.13	80	0.55	102	0.5	0.12 (0.03- 0.57)	0.0021	0.16 (0.03-0.71)	0.0069
	C/G	13	0.87	54	0.37	84	0.41	12.86 (2.68-61.70)	<0.0001	9.28 (2.01-42.8)	5×10^{-4}
	G/G	0	-	11	0.08	20	0.1		-	--	-
IL 1A rs1800587	C/C	7	0.47	62	0.43	100	0.49	1.17 (0.40-3.40)	0.790	0.93 (0.32-2.66)	1.00
	C/T	8	0.53	50	0.34	87	0.42	2.08 (0.70-6.20)	0.189	1.65 (0.56-4.80)	0.36
	T/T	0	-	33	0.23	19	0.09	--	-	--	-
IL1B rs16944	G/G	8	0.53	57	0.39	87	0.42	1.76 (0.61-5.13)	0.408	1.56 (0.55-4.47)	0.429
	G/A	7	0.47	65	0.45	98	0.48	1.08 (0.37-3.18)	0.891	0.84 (0.29-2.45)	0.75
	A/A	0	-	23	0.16	21	0.1	--	-	--	-
IL1B rs1143634	C/C	9	0.60	72	0.50	108	0.52	1.52 (0.51-4.49)	0.589	1.36 (0.47-3.96)	0.604
	C/T	6	0.40	52	0.36	81	0.39	1.38 (0.45-4.22)	0.571	1.08 (0.36-3.21)	0.89
	T/T	0	-	21	0.14	17	0.08	--	-	--	-

Number (N.) frequency (Freq.) odd ratio (OR) and 95% Confidence Interval (95% CI) have been reported.

* Dominant model: ARDS Vs w/oARDS OR: 10.99 (2.29-52.69) p<0.0003; ARDS Vs Controls OR: 6.23 (1.35-28.74) p = 0.0054.

Table S2. Analysis of SNP frequencies in patients affected by Mediterranean Spotted Fever (MSF) complicated with Neurologic symptoms (N-Sympt) compared to MSF without N-Sympt (No- N-Sympt) and Healthy Controls (adjusted by 64 years Age cut off and Gender).

**Genes and SNP Alleles**		**Ne-Sympt**		**No- Ne-Sympt**		**Controls**		**NeSympt Vs No-Ne-Sympt**		**Ne-Sympt Vs Controls**	
		**N.**	**Freq.**	**N.**	**Freq.**	**N.**	**Freq.**	**OR (95% CI)**	***p* Value**	**OR (95% CI)**	***p* Value**
TIRAP rs8177374	C/C	19	0.91	115	0.83	178	0.86	1.98 (0.43-9.09)	0.532	1.49 (0.33-6.77)	1.00
	C/T	2	0.09	21	0.15	22	0.11	0.61 (0.13-2.86)	0.51	1.05 (0.22-5.03)	0.95
	T/T	0	0	3	0.02	6	0.03	--	--	--	--
IL-18 rs187238	C/C	10	0.48	72	0.52	102	0.5	0.85 (0.34-2.12)	0.846	0.93 (0.38-2.28)	1.00
	C/G	9	0.43	58	0.42	84	0.41	1.11 (0.43-2.87)	0.83	1.03 (0.41-2.60)	0.95
	G/G	2	0.09	9	0.06	20	0.1	1.30 (0.25-6.63)	0.76	0.89 (0.19-4.21)	0.88
IL 1A rs1800587	C/C	7	0.333	62	0.45	100	0.49	0.62 (0.24-1.63)	0.357	0.53 (0.21-1.37)	0.252
	C/T	7	0.333	51	0.37	87	0.42	0.79 (0.29-2.11)	0.63	0.68 (0.26-1.79)	0.43
	T/T	7	0.333	26	0.19	19	0.09	2.47 (0.87-6.96)	0.098	6.03 (2.00-18.19)	0.0024
*IL1B * rs16944*	G/G	3	0.14	62	0.44	87	0.42	0.21 (0.06- 0.73)	0.0085	0.23 (0.07-0.79)	0.0173
	G/A	18	0.86	54	0.39	98	0.48	9.83 (2.74-35.29)	<0.0001	6.03 (1.71-21.33)	0.0011
	A/A	0	0	23	0.17	21	0.1	--	--	--	--
IL1B rs1143634	C/C	12	0.57	69	0.50	108	0.52	1.35 (0.54-3.42)	0.641	1.21 (0.49-2.99)	0.819
	C/T	4	0.19	54	0.39	81	0.39	0.39 (0.12-1.22)	0.083	0.36 (0.12-1.13)	0.059
	T/T	5	0.24	16	0.11	17	0.08	2.40 (0.76-7.58)	0.16	3.56 (1.11-11.36)	0.046

Number (N.) frequency (Freq.) odd ratio (OR) and 95% Confidence Interval (95% CI) have been reported.

* N-Sympt Vs No- N-Sympt Dominant model (A/G-A/A Vs G/G) OR:5.02 (1.40-17.98), p = 0.0043; N-Sympt y Vs Controls Dominant model (A/G-A/A Vs G/G) OR: 3.82 (1.08-13.57) p = 0.019.

Table S3. Analysis of SNP frequencies in patients affected by Mediterranean Spotted Fever (MSF) complicated with Sepsis compared to MSF without Sepsis (no-Sepsis) and Healthy Controls (adjusted by 64 years Age cut off and Gender).

Genes and SNP Alleles		Sepsis		No- Sepsis		Controls		Sepsis Vs No- Sepsis		Sepsi Vs Controls	
		N.	Freq.	N.	Freq.	N.	Freq.	OR (95% CI)	*p* Value	OR (95% CI)	*p* Value
TIRAP rs8177374	C/C	14	0.82	120	0.84	178	0.86	0.89 (0.24-3.36)	1.00	0.73 (0.20-2.72)	0.713
	C/T	3	0.18	20	0.14	22	0.11	1.40 (0.36-5.38)	0.64	2.19 (0.55-8.76)	0.29
	T/T	0	0	3	0.02	6	0.03	--	-	--	-
IL-18 rs187238	C/C	9	0.53	73	0.51	102	0.5	1.08 (0.39-2.95)	1.00	1.15 (0.43-3.09)	0.807
	C/G	7	0.41	60	0.42	84	0.41	1.05 (0.37-3.00)	0.92	0.91 (0.33-2.56)	0.87
	G/G	1	0.06	10	0.07	20	0.1	0.69 (0.08-5.88)	0.72	0.54 (0.07-4.37)	0.53
IL 1A rs1800587	C/C	5	0.30	64	0.45	100	0.49	0.51 (0.17-1.54)	0.303	0.44 (0.15-1.30)	0.205
	C/T	6	0.35	52	0.36	87	0.42	0.86 (0.29-2.50)	0.78	0.75 (0.26-2.14)	0.59
	T/T	6	0.35	27	0.19	19	0.09	2.75 (0.90-8.42)	0.087	6.97 (2.09-23.20)	0.0027
IL1B rs16944	G/G	3	0.18	62	0.43	87	0.42	0.28 (0.08-1.01)	0.065	0.29 (0.08-1.05)	0.069
	G/A	14	0.82	58	0.41	98	0.48	7.04 (1.92-25.82)	<0.0007	4.65 (1.28-16.88)	0.0087
	A/A	0	0	23	0.16	21	0.1	--	-	--	-
IL1B rs1143634	C/C	10	0.59	71	0.50	108	0.52	1.45 (0.52-4.02)	0.609	1.30 (0.50-3.54)	0.801
	C/T	2	0.12	56	0.39	81	0.39	0.21 (0.05-0.96)	0.019	0.21 (0.05-0.96)	0.018
	T/T	5	0.29	16	0.11	17	0.08	3.42 (1.04-11.26)	0.056	4.75 (1.43-15.84)	0.018

Number (N.) frequency (Freq.) odd ratio (OR) and 95% Confidence Interval (95% CI) have been reported.

Table S4. Analysis of SNP frequencies in patients affected by Mediterranean Spotted Fever (MSF) complicated with Septic shock (Sept-Sh) compared to MSF without Sept-Sh (no- Sept-Sh) and Healthy Controls (adjusted by 64 years Age cut off and Gender).

**Genes and SNP Alleles**		**Sept-Sh**		**No- Sept-Sh**		**Controls**		**Sept-Sh Vs No-Sept-Sh**		**Sept-Sh Vs Controls**	
		**N.**	**Freq.**	**N.**	**Freq.**	**N.**	**Freq.**	**OR (95% CI)**	***p* Value**	**OR (95% CI)**	***p* Value**
TIRAP rs8177374	C/C	13	0.87	121	0.84	178	0.86	1.29 (0.273-6.09)	1.00	1.02 (0.22- 4.78)	1.00
	C/T	2	0.13	21	0.14	22	0.11	0.96 (0.20-4.67)	0.96	1.68 (0.33-8.52)	0.55
	T/T	0	-	3	0.02	6	0.03	--	--	--	--
IL-18 rs187238	C/C	7	0.47	75	0.52	102	0.5	0.82 (0.28-2.37)	0.789	0.89 (0.31-2.55)	1.00
	C/G	7	0.47	60	0.41	84	0.41	1.41 (0.47-4.22)	0.54	1.14 (0.39-3.35)	0.81
	G/G	1	0.06	10	0.07	20	0.1	0.77 (0.09-6.66)	0.81	0.61 (0.07-5.02)	0.62
*IL 1° * rs1800587*	C/C	3	0.20	66	0.45	100	0.49	0.30 (0.08- 1.11)	0.098	0.26 (0.07-0.97)	0.0351
	C/T	6	0.40	52	0.36	87	0.42	1.05 (0.35-3.18)	0.93	0.92 (0.31-2.74)	0.88
	T/T	6	0.40	27	0.19	19	0.09	3.65 (1.13-11.77)	0.036	9.25 (2.59-33.01)	0.001
IL1B # rs16944	G/G	2	0.13	63	0.44	87	0.42	0.20 (0.04-0.92)	0.027	0.21 (0.05-0.96)	0.03
	G/A	13	0.87	59	0.41	98	0.48	9.86 (2.12-45.75)	0.0004	6.29 (1.37-28.98)	0.0051
	A/A	0	-	23	0.16	21	0.1	--	--	--	--
IL1B rs1143634	C/C	8	0.53	73	0.50	108	0.52	1.13 (0.3-3.27)	1.00	1.04 (0.36-2.97)	1.00
	C/T	2	0.13	56	0.39	81	0.39	0.25 (0.05-1.08)	0.087	0.24 (0.05-1.13)	0.054
	T/T	5	0.34	16	0.11	17	0.08	4.27 (1.25-14.61)	0.029	5.84 (1.68-18.14)	0.0097

Number (N.) frequency (Freq.) odd ratio (OR) and 95% Confidence Interval (95% CI) have been reported.

* Sept-Sh Vs Controls Dominant model (G/C-C/C Vs G/G) OR: 4.23 (1.13-15.85), p = 0.018.

# Sept-Sh Vs No- Sept-Sh Dominant model (G/C-C/C Vs G/G) OR: 5.19 (1.12-24.04) p = 0.014; Sept-Sh Vs Controls Dominant model (G/C-C/C Vs G/G) OR: 3.92 (0.85-18.13) p = 0.046.

Table S5. Analysis of SNP frequencies in patients affected by Mediterranean Spotted Fever (MSF) complicated with Coma compared to MSF without Coma (no-Coma) and Healthy Controls (adjusted by 64 years Age cut off and Gender).

**Genes and SNP Alleles**		**Coma**		**No-Coma**		**Controls**		**Coma Vs No-Coma**		**Coma Vs Controls**	
		**N.**	**Freq.**	**N.**	**Freq.**	**N.**	**Freq.**	**OR (95% CI)**	***p* Value**	**OR (95% CI)**	***p* Value**
TIRAP rs8177374	C/C	6	0.75	128	0.84	178	0.86	0.56 (0.11-2.96)	0.617	0.47 (0.09-2.46)	0.316
	C/T	2	0.25	21	0.14	22	0.11	2.18 (0.41-11.69)	0.39	3.28 (0.58-18.41)	0.21
	T/T	0	0	3	0.02	6	0.03	-	--	--	--
IL-18 rs187238	C/C	3	0.37	79	0.52	102	0.50	0.55 (0.13-2.40)	0.487	0.612 (0.14-2.63)	0.722
	C/G	5	0.63	62	0.41	84	0.41	2.56 (0.57-11.50)	0.31	2.27 (0.52-9.95)	0.27
	G/G	0	-	11	0.07	20	0.10	--	--	--	--
IL 1A rs1800587	C/C	0	-	69	0.45	100	0.49	--	--	--	--
	C/T	5	0.63	53	0.35	87	0.42	2.99 (0.68-13.27)	0.14	2.36 (0.54-10.28)	0.24
	T/T	3	0.37	30	0.20	19	0.09	2.68 (0.58-12.30)	0.22	9.00 (1.75-46.30)	0.015
IL1B rs16944	G/G	1	0.13	64	0.42	87	0.42	0.19 (0.02-1.64)	0.144	0.195 (0.02-1.62)	0.145
	G/A	7	0.87	65	0.43	98	0.48	9.52 (1.14-79.70)	0.0096	6.90 (0.83-57.64)	0.03
	A/A	0	-	23	0.15	21	0.10	--	--	--	--
IL1B rs1143634	C/C	5	0.62	76	0.50	108	0.52	1.67 (0.38-7.22)	0.720	1.51 (0.35-6.49)	0.725
	C/T	1	0.13	57	0.37	81	0.39	0.25 (0.03-2.15)	0.15	0.23 (0.03-1.91)	0.11
	T/T	2	0.25	19	0.13	17	0.08	2.24 (0.41-12.13)	0.38	4.06 (0.73-22.48)	0.15

Number (N.) frequency (Freq.) odd ratio (OR) and 95% Confidence Interval (95% CI) have been reported.
